# The contribution of health behaviours to waist circumference change following employment transitions

**DOI:** 10.1093/eurpub/ckac131.210

**Published:** 2022-10-25

**Authors:** ACT Tam, RA Murphy, W Zhang, AI Conklin

**Affiliations:** 1 School of Population and Public Health, Faculty of Medicine, University of British Columbia, Vancouver, Canada; 2 Cancer Control Research, BC Cancer, Vancouver, Canada; 3 CHÉOS, Providence Research, Vancouver, Canada; 4 Faculty of Pharmaceutical Sciences, University of British Columbia, Vancouver, Canada

## Abstract

**Background:**

Mechanisms through which retirement and later-life job loss lead to subsequent weight change are poorly understood, and include changes to one’s health behaviours (HBs) after employment changes. Our study assessed the potential role of HBs in the impact of employment transitions (ET) on waist circumference (WC), by gender.

**Methods:**

We used two waves of survey data from a Canadian sample of 45- to 85-year-olds with objectively measured WC. For 10,117 participants who were working at baseline, we categorized them into three ET statuses: stayed working, entered retirement, and stopped working. Changes in HBs [sleep, smoking, drinking, and physical activity (PA)] were coded by comparing baseline and follow-up responses. Change in WC was analyzed using multivariable linear regression and multinomial logistic regression models (≥5% gain or loss, no change).

**Results:**

Multivariable models showed that the addition of change in HBs did not alter the effect sizes of ETs on WC change. Regardless of ET status, women who quit smoking had an increased WC compared to persistent non-smokers (1.43cm, 95% Confidence Interval 0.07 - 2.79). Women who became habitual drinkers showed more increases in WC compared to non-habitual drinkers (1.43cm, 0.16 - 2.69). Changes to sleep duration were not associated with WC change; however, women who became satisfied with their sleep had greater WC increases compared to already satisfied sleepers (0.79cm, 0.12 - 1.46). Men who increased their PA by > 1hr to 2hrs were less likely to gain ≥5% weight compared to men with no PA change (OR = 0.75, 0.57 - 0.99). Women who increased their PA by > 1hr to 2hrs were less likely to lose ≥5% weight (OR = 0.74, 0.56 - 0.99).

**Conclusions:**

Our study of this Canadian cohort of middle-aged and older adults suggests some HBs are independent risk factors for weight change rather than mechanisms in the employment-anthropometry relationship.

**Key messages:**

• Health behaviour changes in middle-aged and older adults have anthropometric effects that differ by sex/gender, independent of employment transitions.

• Promotion of persistently good sleep quality to women may help maintain waist size.

